# Event-Based Tone Mapping for Asynchronous Time-Based Image Sensor

**DOI:** 10.3389/fnins.2016.00391

**Published:** 2016-08-31

**Authors:** Camille Simon Chane, Sio-Hoi Ieng, Christoph Posch, Ryad B. Benosman

**Affiliations:** ^1^Pixium VisionParis, France; ^2^Institut National de la Santé et de la Recherche Médicale UMRI S 968, Sorbonne Universités, UPMC Univ Paris 06, UMR S 968, Centre National de la Recherche Scientifique, UMR 7210, Institut de la VisionParis, France

**Keywords:** neuromorphic vision, HDR imaging, tone mapping, silicon retina, AER

## Abstract

The asynchronous time-based neuromorphic image sensor ATIS is an array of autonomously operating pixels able to encode luminance information with an exceptionally high dynamic range (>143 dB). This paper introduces an event-based methodology to display data from this type of event-based imagers, taking into account the large dynamic range and high temporal accuracy that go beyond available mainstream display technologies. We introduce an event-based tone mapping methodology for asynchronously acquired time encoded gray-level data. A global and a local tone mapping operator are proposed. Both are designed to operate on a stream of incoming events rather than on time frame windows. Experimental results on real outdoor scenes are presented to evaluate the performance of the tone mapping operators in terms of quality, temporal stability, adaptation capability, and computational time.

## 1. Introduction

The human visual system can perceive a dynamic range of 160 dB overall (Hood, 1998), discerning details both in bright sunlight and in dim starlight. The intra-scene dynamic range of the human eye has been reported to exceed 120 dB even in relatively low light conditions. At the same time, the retina can detect flashes as short as a few milliseconds. Designing a camera that matches these characteristics is a challenge.

Commercially available high-speed cameras are routinely used to study fast phenomena. With acquisition rates of tens of kHz and higher, these cameras beat the temporal resolution of the human retina but must usually rely on off-line processing, prohibiting their use for real-time computer vision. In addition, due to short exposure times at high frame rates, they must be operated under very high scene illumination levels. Scenes (or parts of scenes) with lower illumination are not acquired adequately. As a result, the dynamic range of high-speed cameras is limited.

The development of high dynamic range (HDR) image and video acquisition systems is an active research topic. Such acquisition systems generally follow one of two paradigms: In one approach, the image sensors use non-linear compressive transfer characteristics, either physical logarithmic relations or piece-wise linear functions. In the second approach, data from multiple linear captures are combined to obtain a single HDR frame (Yang et al., 1999). Both approaches generally lead to increased complexity in acquiring and processing the raw sensor data, limiting the achievable frame rate and hence the temporal resolution of the acquired image data.

### 1.1. Acquiring HDR data at high temporal resolution

Another less widely used approach to HDR imaging is referred to as “time-to-saturation.” This method encodes the light intensity information as the amount time it takes to collect a given number of photons rather than by how many photons have been collected during a fixed period of time (the exposure time). Unlike the conventional approach where both dimensions, exposure time and full-well capacity, are limited and consequently saturation can happen when the well is full before the end of the exposure time, time-to-saturation capture unlocks the time dimension of time, allowing arbitrary exposure times. Doing this, the dynamic range can increase drastically and is now only limited by how short an amount of time can be measured at the bright end, and by dark current integration at the dark end of the scene. Obviously, this increase in dynamic range is traded in for a decrease in temporal resolution if a scene is (partially) dark and integration takes a long time. Another drawback is the relatively difficult and complex implementation since it is necessary to detect the end of integration point in time separately for each pixel and record and communicate the information off the pixel array.

The field of biologically-inspired neuromorphic vision and image sensing (Posch et al., 2014; Lenero-Bardallo et al., 2015) has been investigating this approach to visual information capture for quite some time and has tackled these problems. E.g., the AER (address event representation) protocol (Lazzaro and Wawrzynek, 1995) developed in this community allows the encoding and reading out of time information from individual pixels efficiently and with high temporal precision.

**Figure 1:**
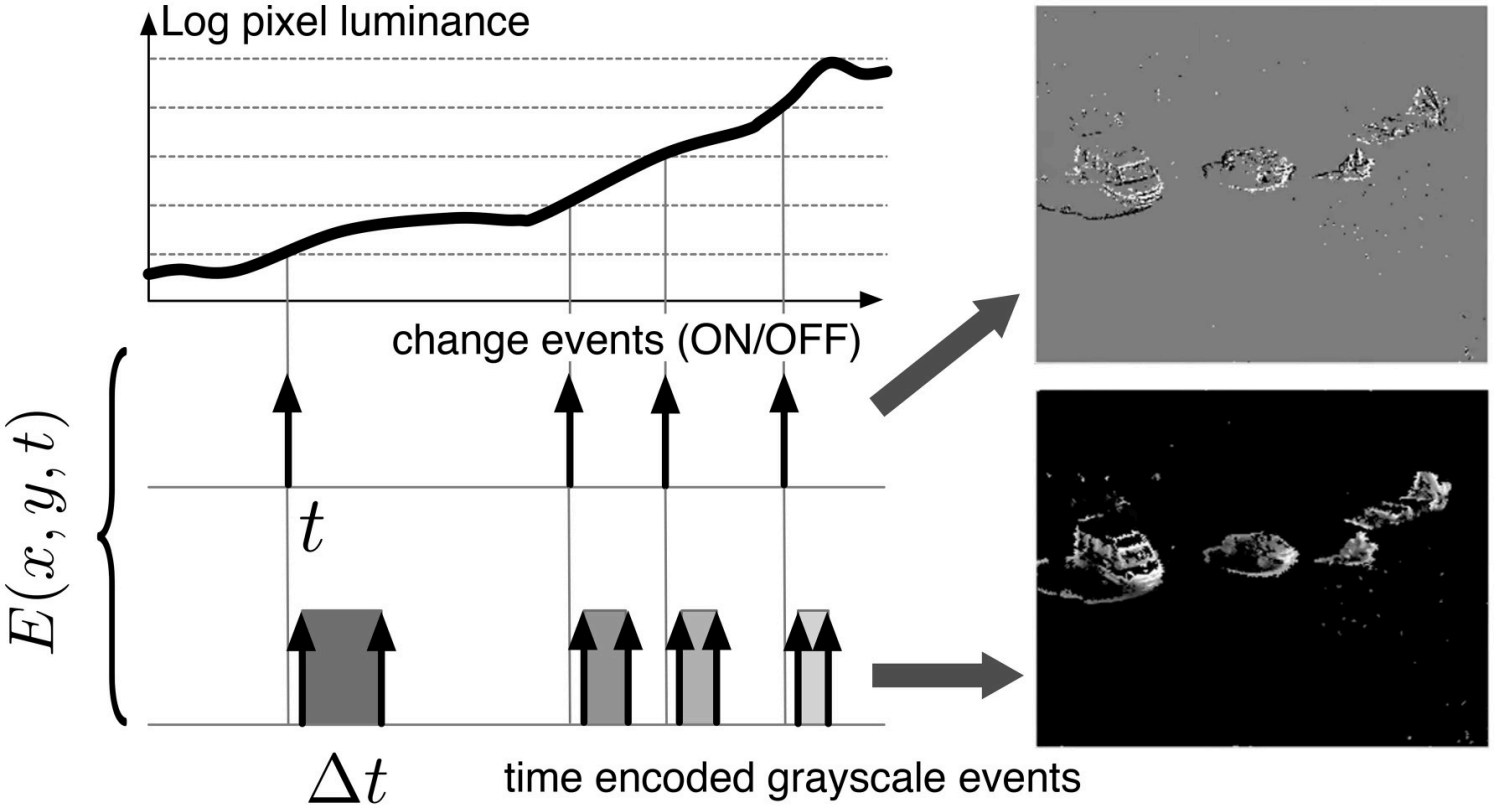
**Functional diagram of the event-based acquisition sensor used in this work**. Two types of asynchronous events, encoding change and brightness information, are generated and transmitted individually by each pixel of the imaging array. Δ*t* is the time difference between the crossing of two thresholds and is inversely proportional to the pixel luminosity. Top right: Change events accumulated over a few milli seconds. Pixels that transmitted an ON event are represented in white, those that transmitted an OFF event are represented in black. The gray background represents pixels for which the luminosity has not changed over the selected time frame. Bottom right: Gray levels are proportional to the inverse of the time difference between two successive events emitted by the exposure detector. Pixels for which the luminosity has not changed over the selected time frame are represented in black.

The asynchronous time-based neuromorphic image sensor (ATIS) proposes a frame-free event-driven auto-sampling way of acquiring exposure information for individual pixels (Posch et al., 2008, 2011). Each fully autonomous pixel combines a relative light intensity level-crossing sampling change detector and a conditional exposure measurement circuit. The change detector initiates the measurement of an exposure/gray-level value when it detects a brightness change of a certain magnitude in its field of view. When triggered by the change detector, the exposure measurement circuit carries out an absolute intensity measurement and encodes the pixel illuminance into the timing of asynchronous pulses using the time-to-saturation approach independently for each pixel (Figure 1). Thanks to the aforementioned time-domain encoding of the exposure information, the dynamic range of the acquired gray-levels is large and exceeds 140 dB for scenes changing relatively slowly.

**Figure 2:**
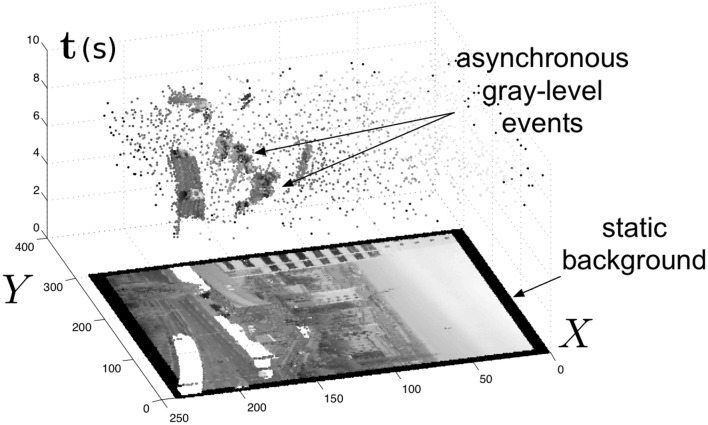
**The spatio-temporal space of imaging events: Static objects and scene background are acquired first**. Then, dynamic objects trigger asynchronous gray-level pixel-events after each change. Frames are absent from this acquisition process.

Another advantage of the per-pixel, event-driven mode of acquisition is the redundancy suppression as data are acquired and transmitted only from the parts of the scene that have changed (Figure 2). Still, the temporal resolution is limited if light levels are low. By using additional information from the fast continuous-time level-crossing sampling circuit, an interpolation scheme that enables intermediate estimations of the instantaneous gray-levels from pixels that receive low light levels from the scene has been proposed (Orchard et al., 2014). This approach provides high temporal resolution data even from dark parts of scenes and permits HDR image data acquisition (>120 dB) at relatively high temporal resolutions—equivalent to kiloframes per second.

### 1.2. Vizualizing HDR data at high temporal resolution

Processing HDR images and video instead of LDR data usually requires minor (or no) changes to the algorithms. However, there are no corresponding high speed, high dynamic range displays to visualize the video data. Conventional displays, monitors or projectors have both a limited dynamic range and a limited display frequency. LCD displays generally advertise a9Dcontrast ratio9D of 1000:1, which is equivalent to a 60 dB dynamic range. CRT displays have an even lower dynamic range of 40 dB. HDR displays are under development (Seetzen et al., 2004) and few are commercially available. Dolby has been developing HDR displays, such as the DR 37P (dynamic range of 100 dB), and they have announced a future product that can reach 120 dB. The display frequency of such displays rarely exceeds 60 Hz.

On the other hand, there are only a few high speed displays available and they are mostly used for research purposes. It has been shown that it is possible to tweak CRT displays to obtain e.g., a 480 Hz refresh rate (Kuroki et al., 2007). Digital Micro-mirror Devices (DMDs) can be used to display binary images at up to 4 kHz. They are capable of displaying gray-level images with a dynamic range of 60 dB, but this reduces the display frequency, since binary pulse-width modulation is used to produce the various shades of gray.

There is increasing evidence that high speed displays are useful and important. E.g., if changes in the visual field are involved, such as with a moving camera, displaying at higher frequencies influences both visual comfort and perceived spatial accuracy. Kuroki et al. (2007) showed that increasing the projection rate improves the perceived quality of a video sequence. They asked subjects to evaluate various video sequences captured at 1000 Hz and projected between 60 and 480 Hz using a custom CRT monitor. They found that the perceived quality is improved by increasing the projected frame rate up to 240 Hz though no improvement has been noticed for higher rates. In addition, preliminary results suggest that a high frequency display (up to 1000 Hz) can compensate for low spatial accuracy.

The visualization of HDR data on LDR display devices requires a compression of the dynamic range that preserves the visual sensation of the scene as faithfully as possible. This process is called tone mapping. The LDR output must preserve visual details and contrasts provided by the HDR data as much as possible, e.g., to simultaneously display details in shadows and in sunlit areas of a scene.

The problem this work addresses is to tone-map the output of an event-based HDR high temporal resolution ATIS imaging sensor (Posch et al., 2011). The tone mapping must be done in real time even at a high event rates. The high event rate is necessary to completely describe scenes presenting a lot of movement, for example when the camera is in motion. The tone mapping must also automatically adapt to unpredictable illumination changes. Real-time processing and display is necessary to enable the adjustment of optical parameters such as focus and zoom.

Existing solutions to the LDR display of HDR video are all frame-based. Adapting frame-based solutions to event-based tone-mapping requires selecting those that are adapted to an event-based formulation. Event-based algorithms are generally incremental: each incoming event is processed independently and few calculations are performed on it. A non greedy event-based algorithm requires few calculations on small neighborhood. The data are then tone-mapped and, depending on the application, displayed at the required frequency. The current focus has not been on the display of event-based data but on its processing for computer vision applications, e.g., for visual motion estimation (Orchard and Etienne-Cummings, 2015), feature tracking (Lagorce et al., 2015), optical flow (Benosman et al., 2012), or machine learning (Perez-Carrasco et al., 2013).

We present a simple algorithm for the tone mapping of an event-based video stream. Our algorithm has been tested on two outdoor scenes taken from the inside of a car. All results are given for an 8-bit output (48 dB). We first present the event-based sensor used in this work, followed by an overview of event-based HDR tone mapping. We then present a global and a local tone mapping algorithm for event-based vision sensors. Finally, we evaluate these operators and compare them to those currently used for the display of event-based acquisitions.

### 1.3. Frame-based HDR tone mapping

The problem of displaying high dynamic range data on low dynamic range displays dates from the advent of HDR photography and the beginning of computer graphics. The main requirements of a satisfying image tone mapping operator is that all features, dark and bright, should be visible simultaneously, while preserving the contrast impression of the visual scene (Duan and Qiu, 2004). Further requirements, given by Krawczyk et al. (2007) are that the tone mapping must provide consistent results despite the vast diversity of natural scenes; it must avoid introducing artifacts such as contrast reversal or black halos; the overall brightness of the output image must be faithful to the context; a daylight scene should be distinguishable from a night scene. Tone mapping operators should also have few, intuitive parameters. They should be fast for interactive and realtime applications.

Until recently, tone mapping operators were developed for static images and were not adapted to the tone mapping of a video stream. In addition to the aforementioned requirements, video tone mapping operators must provide temporal adaptation and coherence. This means that the tone mapping operators must adapt to varying light conditions throughout a video, without introducing sharp jumps in scene luminosity nor flickering. They must also retain the same visual sensation throughout the video. Ideally we want algorithms with no manual parameters, otherwise it is generally difficult to fix a parameter that is satisfactory over a video stream where the scene can vary greatly and unpredictably.

Tone mapping operators are generally classified in two categories: global (or spatially uniform) vs. local (or spatially varying) (DiCarlo and Wandell, 2000; Krawczyk et al., 2007). In the global case, a single monotonic function, the Tone Reproduction Curve (TRC), is applied to the full image (or successive frames). In this case, a given input value is always mapped to the same output gray-level.

Local algorithms are referred to as tone mapping operators. They mimic the human visual system by locally adapting the dynamic range. They may map two identical input values to different gray-levels, depending on their neighboring input values. These operators are generally more subject to producing artifacts such as halos. Local operators typically require complex computations to obtain a first tone mapped image and possibly even more to remove artifacts. It is thus easier for global operators to satisfy realtime requirements. However, local operators are better adapted to an event-based formulation. Instead of successively processing each subregion of a frame, for each incoming event we process its local neighborhood.

A psychophysical study by Yoshida et al. (2005) compared four global operators (linear scaling and methods by Ward Larson et al., 1997; Pattanaik et al., 2000; Drago et al., 2003) and three local operators (fast bilateral filtering Durand and Dorsey, 2002, as well as methods by Ashikhmin, 2002; Reinhard et al., 2002). They found that global operators are perceived as brighter than local operators and as slightly more contrasted. However, more details are perceived in the bright regions when local operators are used compared to global operators.

We address the case of gray-level tone mapping since this is the type of signal provided by the ATIS, though the great majority of tone mapping operators are developed for color images. The simplest tone mapping operator is the linear scaling of the input values to the output range. The problem with this operator is that it does not adapt to the input signal distribution. It preserves the original contrast but visibility is lost where the input histogram is dense. To limit the influence of noisy extremum pixels, we generally scale without accounting for the top and bottom percentiles by saturating these values to the maximum and minimum output dynamic range values. Many image tone mapping algorithms rely on the use of the maximum scene luminance for scaling. This is generally a problem when adapting these algorithms for the tone mapping of HDR video, since it produces important changes in the overall gray-levels during camera panning. It can be tedious to adjust the percent of data to ignore at the top and bottom of the histogram since this must be done individually for each image. In the case of a video, the ideal values may vary over time. Furthermore, even after fine tuning these parameters, the resulting tone mapped image may be simultaneously overexposed in certain regions and under-exposed in others.

**Figure 3:**
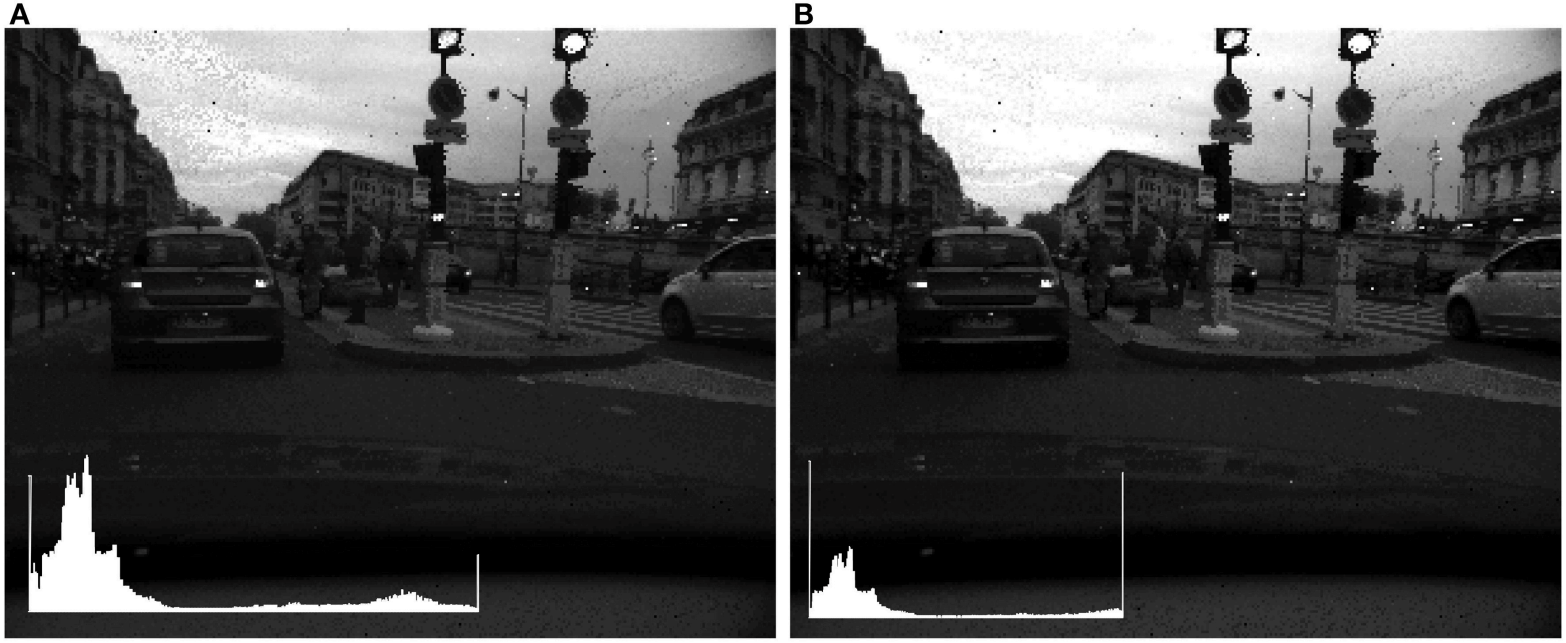
**Linear tone mapping with increasing percentage of high and low values excluded from scaling**. **(A)** 1%. **(B)** 5%. Insets: Image histogram. The different effect of the tone mapping is particularly visible in the sky area.

For example, Figure 3 shows a 140 dB frame linearly tone mapped into a 48 dB signal after setting a fixed percentage (left 5%, right 1%) of the top and bottom dynamic range to 0 and 255. Both images are dark and few details are visible, though in the right image 5% of the brightest pixels are saturated to 255 and 5% of the darkest to 0. We present examples with the same percentage of top and bottom percentage removed, but it is generally best to adjust these parameters independently, even though this results in two parameters to set.

**Figure 4:**
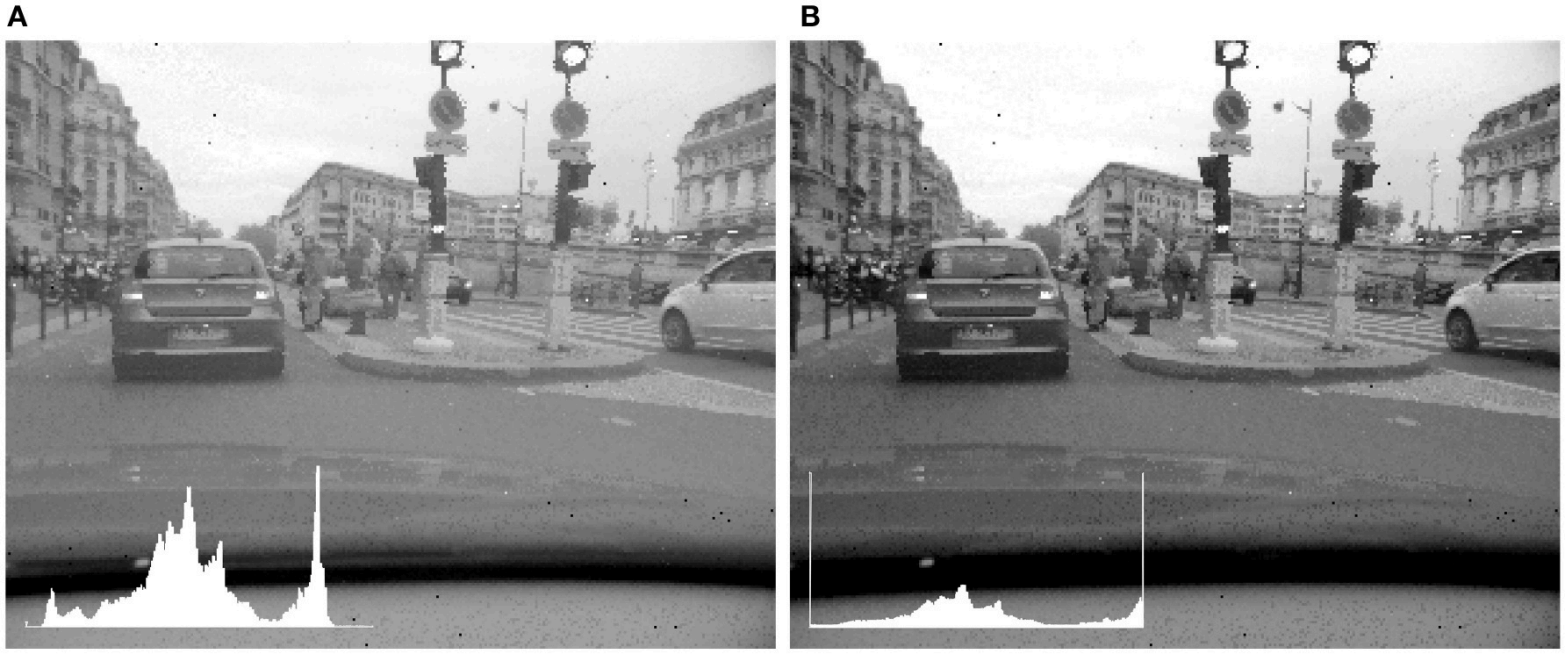
**Logarithmic tone mapping with increasing percentage of high and low values excluded from scaling**. **(A)** 1%. **(B)** 5%. Insets: Image histogam. In addition to an improved contrast in the left image, the difference between the two images is most notable in the representation of the sky and clouds.

Instead of tone mapping the input with a linear function, it is also possible to use a logarithm to map the input values into the output range, possibly removing a percentage of the top and bottom dynamic range. The logarithmic function is well adapted to the display of the data provided by the sensor used in this work, which is sensitive to changes in log intensity. The need to manually select which portion of the histogram to ignore remains. However, this parameter is much easier to adjust for the logarithmic function than for the linear function. Previously, in the case of the linear tone mapping, excluding the top and bottom 5% of the dynamic range resulted in an image that was both saturated and dark. In the case of the logarithmic function, illustrated Figure 4, both images provide a satisfactory tone mapping, though in one case the top and bottom 1% of the histogram is respectively tone mapped to 0 and 255, while in the second case 5% of the top and bottom of the histogram is tone mapped to 0 and 255. A tone mapping operator which uses different log-bases was defined by Drago et al. (2003) for the tone mapping of images, though there is a parameter to adjust for optimal results.

Histogram equalization is another simple tone mapping operator. However, the resulting image is unrelated to the physical brightness and contrast. In particular, this operator can greatly exaggerate contrast. The histogram-based method developed by Ward Larson et al. (1997) prevents display contrast from exceeding scene contrast but does not prevent the display from having too low contrast. Qiu and Duan (2005) and Duan and Qiu (2004) developed a tone mapping operator that slides between linear scaling and histogram equalization, depending on the input parameter. They also define a local operator (Duan et al., 2010) based on this global operator. Mantiuk et al. (2006) also developed a method based on histogram equalization. In this type of approach, computation time increases as the output dynamic range increases.

### 1.4. Event-based tone mapping

Reinhard developed an image tone mapping operator inspired by common photographic practice. The global “initial luminance mapping” is refined by an automatic dodging and burning. Based on a psychophysical study (Ledda et al., 2005), this tone mapping operator performs well on a variety of scenes and particularly for gray-level images, as is our case. We present an adaptation of Reinhard's “initial luminance mapping” (Reinhard et al., 2002) for the tone mapping of event-based acquisitions. Reinhard also defined a method to automatically fix the two parameters for the initial global tone mapping and to determine whether the local dodging and burning is necessary (Reinhard, 2002). Kiser and Reinhard (2012) define a frame-based video tone mapping operator based on the global initial tone mapping using the automatic parameter settings and a leaky integrator to avoid video flickering. Compared to Reinhard's “Initial luminance mapping,” the tone mapping operators presented here set the “key value” to *a* = 1 and define the maximum scene luminance as infinity. This tone mapping operator is suitable for an asynchronous i.e., an event-based formulation.

## 2. Materials and methods

### 2.1. A global operator for event-based tone mapping

Of the two asynchronous event streams, we only process the brightness events. Let *E*(*x, y, t*) = [*x, y, t*, Δ*t*]^*T*^ be a quadruplet giving the pixel position [*x, y*]^*T*^, the time *t* of the event, and the time difference Δ*t*, inversely proportional to the pixel luminosity (see Figure 1). The output display values **D**(*x, y*) are calculated directly from the time stamp differences Δ*t*(*x, y*), as explained by algorithm 1.

**Algorithm 1:** Global event-based tone mapping

Δ*t*_min_ = 10^9^, an arbitrarily large value
**for all** *E*(*x, y, t*) **do**
**T**(*x, y*) ← Δ*t*
if Δ*t* < Δ*t*_*min*_ **then**
Δ*t*_*min*_ ← Δ*t*
*x*_*min*_ ← *x*
*y*_*min*_ ← *y*
calculate *G* using equation 1
update **D** using equation 3
**else if** *x* = *x*_*min*_ and *y* = *y*_*min*_ **then**
Δ*t*_*min*_ ← min(**T**)
(*x*_*min*_, *y*_*min*_) ← arg min(**T**)
calculate *G* using equation 1
update **D** using equation 3
**else**
$\text{D}\left(x,y\right)\leftarrow \frac{1}{1+\frac{\text{T}\left(x,y\right)}{G}}$
** end if**
**end for**

These time stamp differences are stored in matrix **T**(*x, y*). Whenever the minimum Δ*t* in the image changes—that is, every time the maximum scene luminance changes—we calculate the geometric mean (or log-average) *G* of the last time differences for every pixel of the signal:

(1) $$G(T)=\sqrt[{X\cdot Y}]{\prod_{x,y}^{X,Y}T(x,y)}=\exp\left(\frac{1}{X\cdot Y}\sum_{x,y}^{X,Y}\log\left(T(x,y)\right)\right),$$

where *X* and *Y* are respectively the width and height of the image. In practice *G* is calculated for non-null values of Δ*t*. This simultaneously protects from a division by zero in the next step and enables us to only take into account pixels where a pair of events has been detected. *G* gives us an estimate of the brightness of the scene. We use *G* to scale the input time differences:

(2) $$S(x,y)=\frac{T(x,y)}{G}.$$

Incoming events are converted to their display value *D* using:

(3) $$D(x,y)=\frac{1}{1+S(x,y)}=\frac{1}{1+\frac{T(x,y)}{G}}.$$

**Figure 5:**
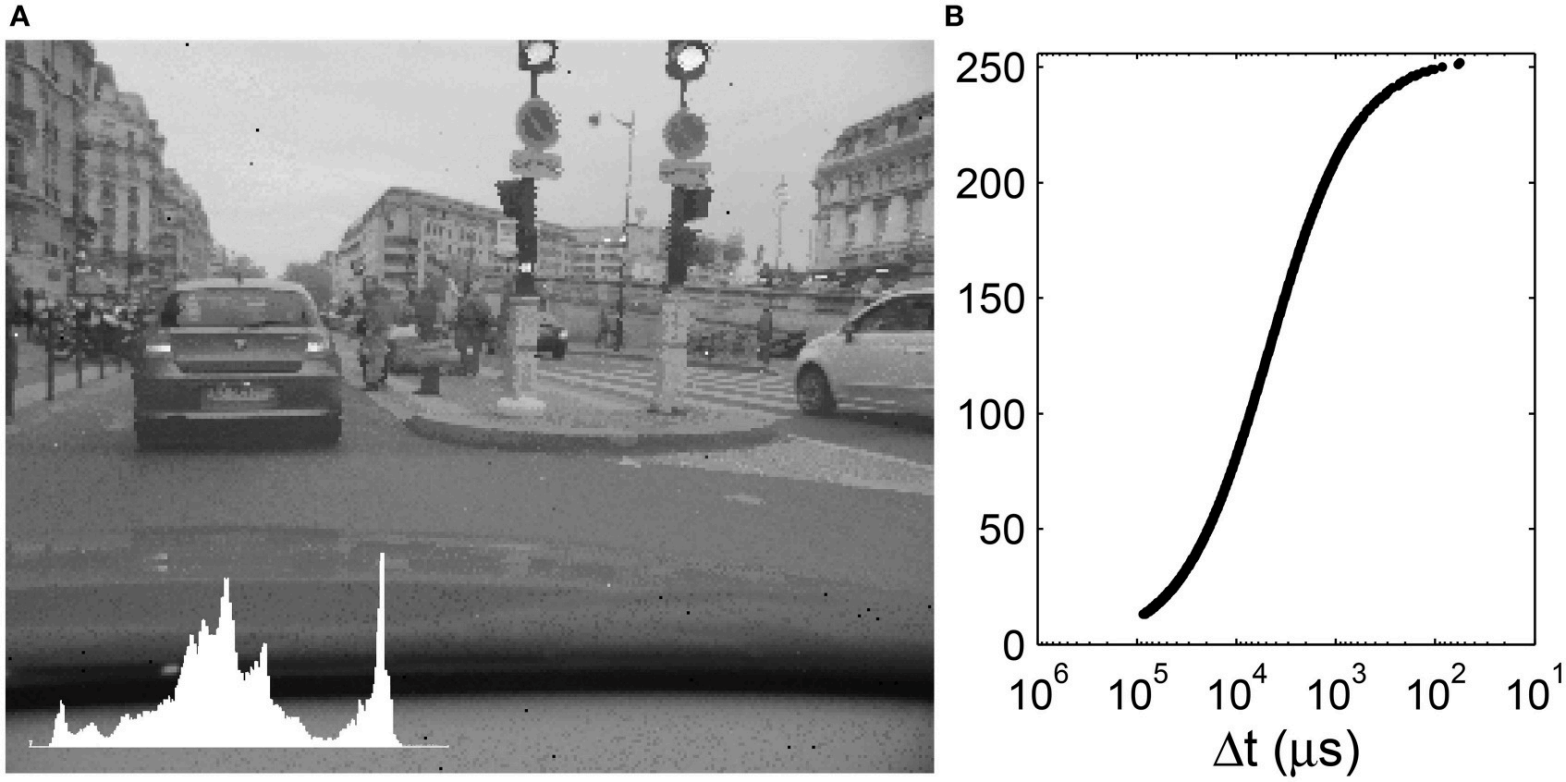
**(A)** Global tone mapping of a 140 dB input to 256 gray-levels. Inset: Image histogram. **(B)** Corresponding tone mapping function. The input time differences are plotted on an inverse logarithmic scale while the output gray-levels are plotted linearly.

This function maps positive values to [0, 1]. The result can then be linearly scaled to the full display range, in our case [0, 255], simply by multiplying the output display values by the maximum output value and rounding the result. This tone reproduction curve is S-shaped and *D*(*x, y*) is set to *D*(*x, y*) = 0.5 if Δ*t* = *G*. Bright events, those for which Δ*t*<*G*, are mapped to [0.5, 1]. Since the geometric mean is lower than the arithmetic mean, very bright events are compressed to a reduced range. On the other hand “medium-luminance” events are logarithmically tone-mapped. When we recalculate *G*, we also update all display values using (Equation 3). The 140 dB reference frame is shown tone-mapped using this global operator in Figure 5. The position of the curve on the abscissa depends on the current value of *G*.

Every change in the minimum Δ*t* thus results in calculating a geometric mean over the full image, which becomes ever more computationally expensive as the size of the sensor increases. Furthermore, this update is sometimes done “uselessly” as a change in the minimum Δ*t* can have a very small impact on the geometric mean and output pixel values.

### 2.2. A local operator for event-based tone mapping

Based on this global operator, we define a local operator described by algorithm 2. Every incoming event is tone-mapped depending on the values of its neighbors. As previously we store the the time-stamped differences in **T**(*x, y*). For every incoming event we compute *G*_*R*_, the geometric mean of the last values of Δ*t* for all pixels within a radius of *R* using the Chebyshev distance:

(4) $$G_{R}(x_{i},y_{i})=\exp\left(\frac{1}{{\left(2R+1\right)}^{2}}\sum_{x\;=\;x_{i}-R}^{x_{i}\;+\;R}\sum_{y\;=\;y_{i}\;-\;R}^{y_{i}\;+\;R}\log\left(T(x,y)\right)\right).$$

**Algorithm 2:** Local event-based tone mapping operators

**for all** *E*(*x, y, t*) **do**
**T**(*x, y*)←Δ*t*
calculate *G*_*R*_ using equation 4
$\text{D}\left(x,y\right)\leftarrow \frac{1}{1+\frac{\Delta t}{G_{R}}}$
**end for**

The display value is calculated, as previously, after normalizing the input values by *G*_*R*_.

**Figure 6:**
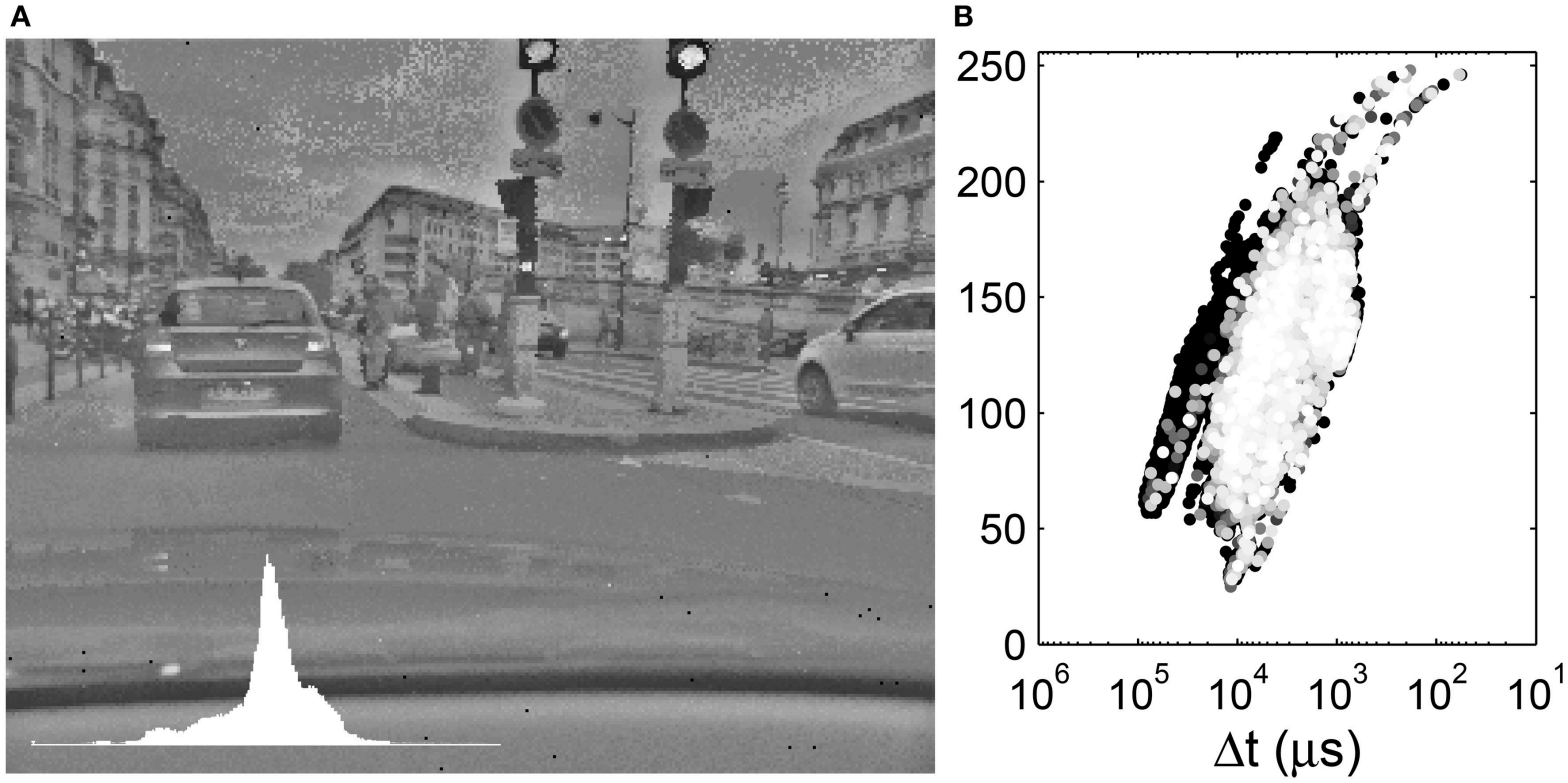
**(A)** Local tone mapping with *R* = 10. Inset: Image histogram. **(B)** Display values as a function of the input time differences. The input time differences are plotted on an inverse logarithmic scale while the output gray-levels are plotted linearly. The 10% most recent events are colored with a gray-level.

**Figure 7:**
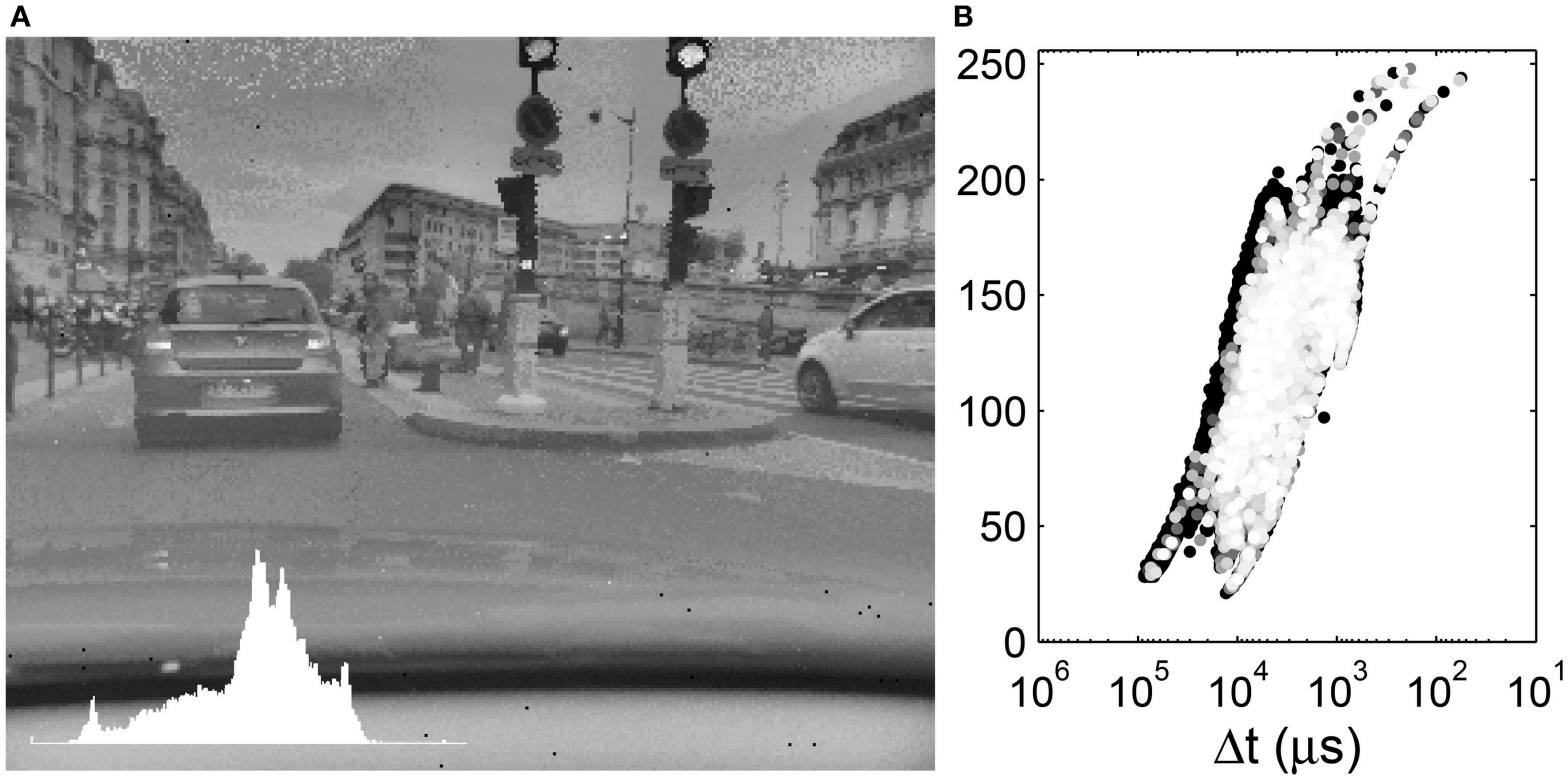
**(A)** Local tone mapping with *R* = 30. Inset: Image histogram. **(B)** Display values as a function of the input time differences. The input time differences are plotted on an inverse logarithmic scale while the output gray-levels are plotted linearly. The 10% most recent events are colored with a gray-level.

Figures 6, 7 show a frame tone mapped using two different radii *R*. Compared to the global tone mapping operator, the local operator introduces a speckle-like noise, especially for low values of *R*. This noise is due to the enhanced local contrast which makes the acquisition sensor noise more visible. In particular the asynchronous pixel update can cause certain pixels that have been tone-mapped earlier on to neighbor more-recently updated pixels. The smaller *R*, the closer Δ*t*_*i*_ is to *G*_*R*_, and thus **D**(*x*_*i*_, *y*_*i*_) tends toward 0.5. With a small radius, the histogram is thus strongly centered around 128. As *R* increases, we increase the contrast in the tone-mapped image.

The local tone mapping introduces a halo effect, mostly visible on the edges of the buildings in our examples. Near the border of the buildings, there is an equal contribution of the light sky and the dark buildings to the geometric mean which increases their relative contrast. For pixels further away from the border, the geometric mean is an average of the luminance of pixels of the same object. This effect is thus more subtle as *R* increases. This is a common artifact of local tone mapping operators, though Durand and Dorsey (2002) designed a local tone mapping operator to avoid such artifacts via an edge-preserving filter.

We clearly see that a given Δ*t* is mapped to different gray levels, depending on its neighboring values. The tone mapping plot is a superposition of curves such as that presented in Figure 5B shifted on the abscissa. As *R* increases, the tone mapping operator becomes more compact: a given value of Δ*t* is mapped to a smaller range of output values. On the other hand, as *R* increases, we tend toward a global operator and the tone mapping operator tends toward a tone mapping function. Increasing the radius size also increases the processing time, since the geometric mean must be computed over a greater number of values. The black background points on the left of the tone mapping plot of Figure 6B represent the car dashboard, which was tone mapped at the beginning of the video and never updated since. An effect of the local tone mapping can be seen in the improved contrast on this dashboard, especially for a small radius such as *R* = 10.

## 3. Results

### 3.1. Test videos

**Figure 8:**
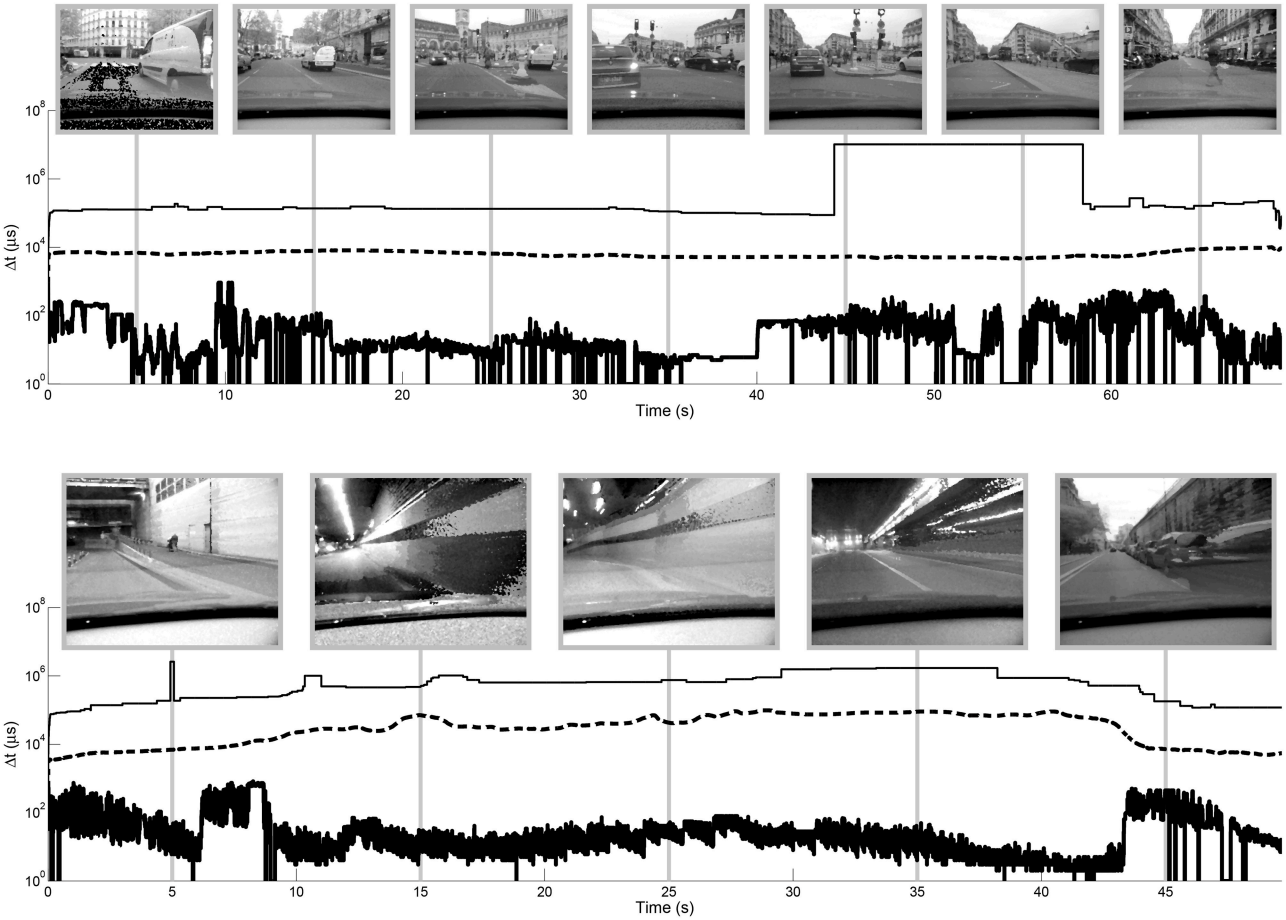
**Evolution of dynamic range of the “city” (top) and “tunnel” (bottom) videos over time**. Maximum, median and minimum time differences are plotted in over time on a semilog axis. Snapshots are shown with logarithmic tone mapping. A spatial median filter is applied to the input data prior to recording the maximum, median and minimum values to remove noise due to unresponsive or over-sensitive pixels (single black and white pixels in examples). The strong discontinuity in the maximum time difference for the “city” video between approx. 45 and 52 s is due to noise that is still present after median filtering on pixels that are infrequently updated. These values are not necessarily distinguishable in the videos since they represent few pixels that may be saturated (in the case of the linear and logarithmic tone mapping) on an equally dark background.

The two algorithms have been tested on several videos. We present in greater detail results from two event-based acquisitions taken from a car: “city” and “tunnel.” The evolution of the dynamic range of these outdoor scenes is illustrated in Figure 8. This figure shows the evolution of minimum, median and maximum luminance of the videos over time. The dynamic range is represented by the area between the top and bottom curves. It is calculated after each incoming event using:

(5) $$\text{DNR}(t)=20\;\log_{10}\frac{\max\;T}{\min\;T}.$$

The median dynamic range is 82 dB for the “city” video and 86 dB for the “tunnel” video, while the maximum dynamic range of these videos is respectively 140 and 119 dB. In both cases the dynamic range is more influenced by the shorter time differences (high luminance) than the longest time differences (low luminance), which varies more smoothly over time. The median value also varies quite smoothly over time. The maximum luminance in these videos seems to be exclusively due to artificial lights (car lights, traffic lights, tunnel lights) and not from the luminosity of the sky. There is thus a greater dynamic range in the tunnel than outside, because of the bright ceiling lights.

### 3.2. Overview

As noted by Yoshida et al. (2005), the global operator is brighter and more contrasted than the local operator. However, some details such as the license plate of the car are perceived more easily in the locally tone mapped images. We presented a few examples of tone-mapped images using the global and local operator. However, since these are video tone mapping algorithms, it is important to see how they fare over time. Three videos are provided as supplementary material. They compare the logarithmic tone mapping, the global operator and the local operator with both *R* = 10 and *R* = 30 on the “tunnel” data, the “city” data and an additional night scene.

The advantage of updating *G* only when the maximum luminance in the scene changes is that it limits the frequency of calculating the geometric mean, which is computationally intensive. As such, less than 1% of the events produce a global update: 0.014 % for the “city” video, 0.12 % for the “tunnel” video. This corresponds to a global update at 71 and 420 Hz respectively, which is still quite high and could be reduced, depending on the display rate.

**Figure 9:**
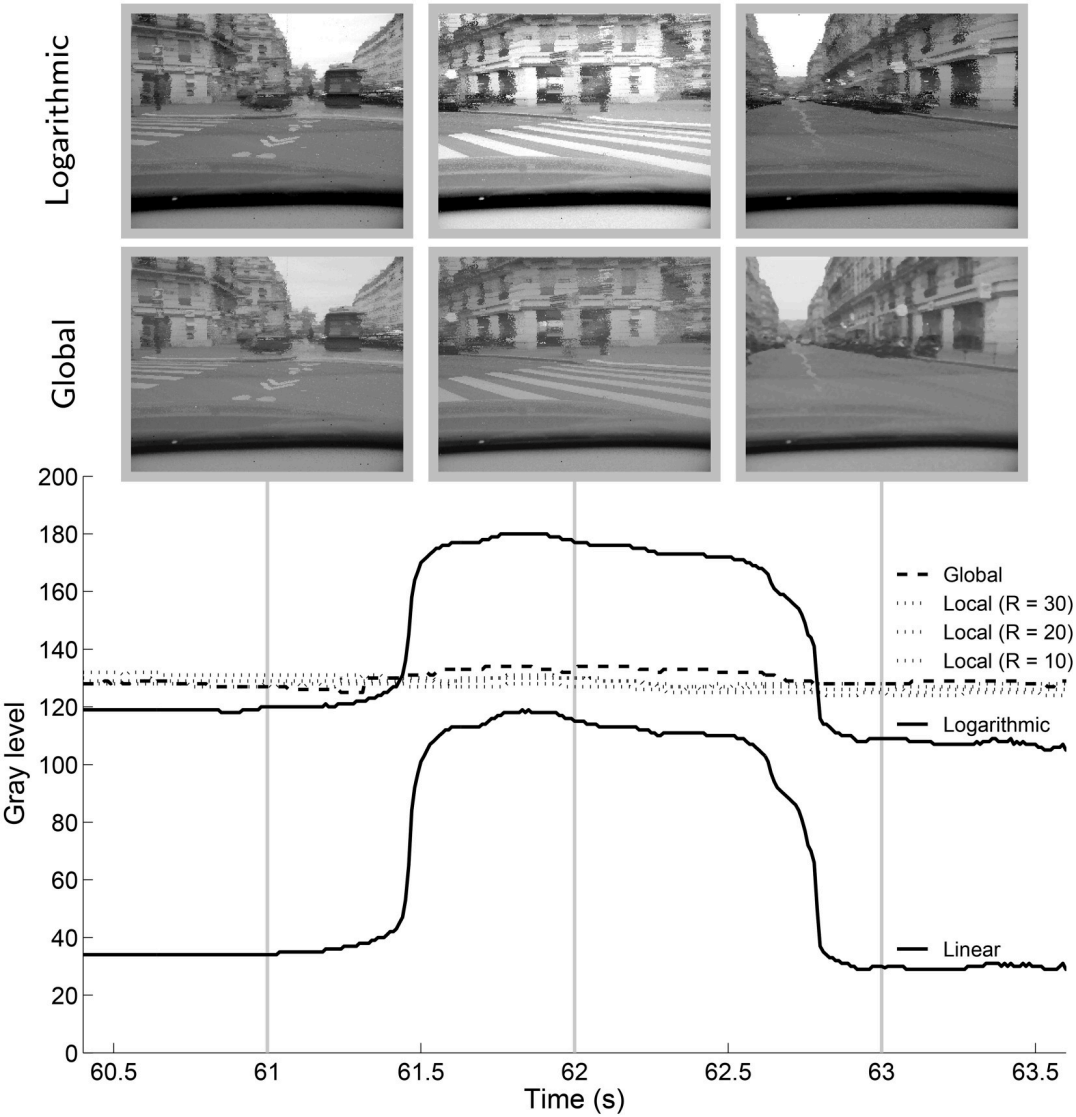
**Median of the gray-levels of for a portion of the “city” video for different tone mapping algorithms**. The linear and logarithmic tone mapping are run with 5% of the top and bottom of the input dynamic range clamped to extremum display values.

Updating *G* only when the maximum luminance changes also permits a smooth adaptation to changes in illumination. If a bright object enters the scene, *G* will be updated several times, as the object appears. The tone mapping function will then be fixed, until the maximum luminosity changes again. Figure 9 shows the evolution of the median of the gray-levels for linear and logarithmic tone mapping compared to the global and local operators presented here for a selected portion of the “city” video. These curves give an idea of the evolution of the luminosity of the tone mapped videos over time. The effect can be observed at 1:00 of supplementary video “city.”

This portion of the video shows the view from the turning car. For a short time (~1 s) the sky is no longer visible in the frame. In the case of the logarithmic or linear tone mapping, the buildings are tone mapped to a lighter value, causing a sudden change in global luminosity. Global and local tone mapping both reduce flicker and the transitions are smoother.

### 3.3. Quality metrics

Yeganeh and Wang (2013) defined a metric to evaluate and compare the quality of image tone mapping operators. The quality index *Q* is based on the combination of a structural fidelity index, *S* and a statistical naturalness index *N*. *S* is based on the widely accepted (Aydin et al., 2008; Mantiuk et al., 2011) Structural Similarity Index Measure (SSIM Wang et al., 2004), a quality metric used to measure the similarity between two images and considered to be correlated with the quality perception of the human visual system. *N* is based on intensity statistics of natural images, it is calculate only on the input LDR image.

We use the default parameters from the implementation of the tone mapping toolbox for Matlab provided by Banterle et al. (2011). The function is simply modified to accept single channel gray-level images, instead of processing the luminance of RGB images.

Table 1 shows the median of the quality index *Q*, of *S* and of *N* for the “city” video tone mapped by several operators every 10 ms. The quality is better using the global tone mapping than the linear and logarithmic mapping. However, the local tone mapping provides a quality index that is lower than the logarithmic quality index, though the quality index of the local tone mapping increases as *R* increases. If we look into the two components of the quality index, we notice that the structural fidelity index is better for the linear, logarithmic and global tone mapping operators than for the local tone mapping, especially as *R* decreases. It is not surprising that *S* is worse for the local tone mapping operator, since it locally modifies the contrast. On the other hand, the statistical naturalness index is much better for the global and local tone mapping operators than for the linear and logarithmic tone mapping operators.

**Table 1:** **Median quality metrics for the “city” video**.

	**Q**	**S**	**N**
Linear	0.83	0.95	0.14
Logarithmic	0.93	0.92	0.75
Global	0.96	0.92	0.91
Local (*R* = 10)	0.86	0.61	0.88
Local (*R* = 20)	0.89	0.69	0.83
Local (*R* = 30)	0.89	0.73	0.79

All the indices are normalized to 1 and increase with the quality.

### 3.4. Computational time

**Figure 10:**
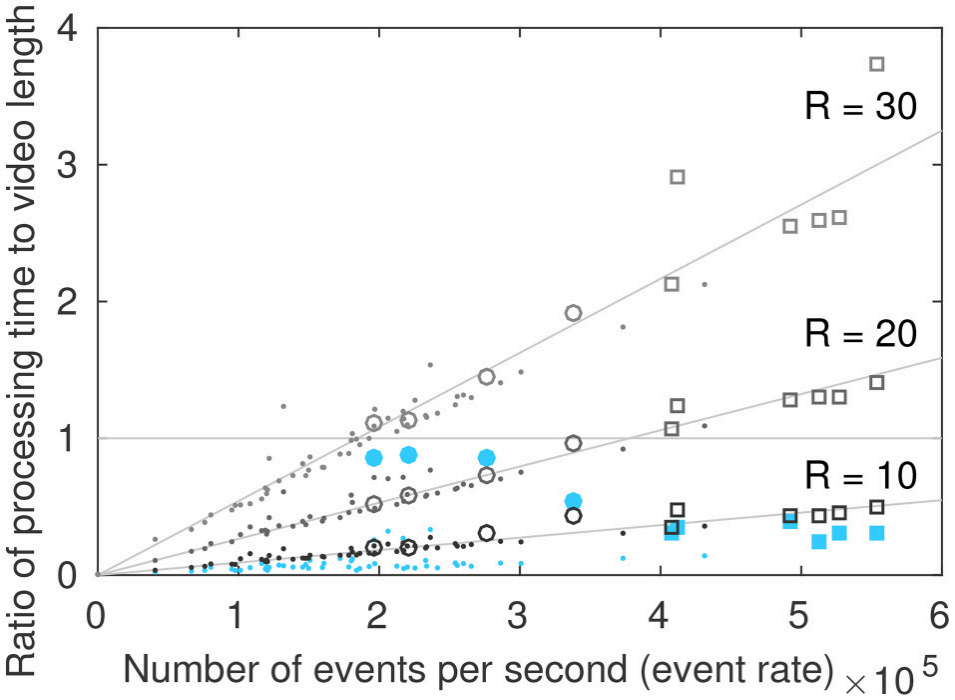
**Processing time as a function of the event rate**. The area below the horizontal line represents real time processing. Squares represent segments of the “city” video, circles represent segments of the “tunnel” video, dots represent other indoor and outdoor scenes. Filled blue circles and squares represent global tone mapping, empty circles and squares represent the local tone mapping for different radius values.

Processing time of the local and global tone mapping is compared in Figure 10. A series of ten indoor and outdoor scenes were divided into 10 s segments and tone mapped. The processing times are given for a C++ implementation on a computer with a 3.1 GHz Intel Core i7 processor. They solely include the event-based tone mapping process and no display procedure.

Scenes acquired with a moving camera, such as the “city” and “tunnel” videos have a higher event rate than those acquired with a static camera. The global algorithm and the local algorithm with *R* = 10 can process an incoming stream of events in real time.

The global tone mapping is not linear as a function of the event rate, since the value of the events directly influences the number of operations to be performed: if the maximum scene luminosity changes, many operations are necessary. The tunnel video is thus the longest to process for a given event rate. This is due to the ceiling lights in the tunnel that successively appear and disappear, changing the maximum luminance of the scene with great frequency and thus causing repeated updates of all pixel values. The maximum value is updated on average every 1 × 105 events for the “tunnel” video, and every 2 × 105 events for the “city” video (median filtered videos). This can be observed on, Figure 8 where the minimum Δ*t* (that is, the maximum luminosity) changes much more frequently in the “tunnel” video than in the “city” video.

For the local algorithm, the processing time linearly increases as the event rate increases. This is not surprising, since a fix set of operations are performed for every incoming event. Though the algorithm can only run in real time for small values of *R* (approximately *R* = 10) in its current implementation, this formulation is adapted for parallel computing. This may enable us to reach real time computing for high speed display for larger values of *R*.

## 4. Discussion

We have presented two algorithms to display event-based HDR acquisitions. Compared to the tone mapping requirements listed Section 1.3, both operators preserve details in the dark and bright regions. Results are consistent for a variety of indoor and outdoor scenes. The contrast impression is better preserved by the global operator than the local operator, which introduces some artifacts, in particular halos. Processing time is independent of the final number of mapping bins. The global algorithm runs in real time, as does the local algorithm for small radii (of the order of *R* = 10). The processing time of the local algorithm is directly proportional to the event rate.

Furthermore, the global algorithm is parameterless while the local algorithm depends on a single parameter, *R*, the radius of the local neighborhood. This parameter trades output quality for processing time and can be set independently from the video content. Finally, it must be outlined that both algorithms provide temporal adaptation and coherence to the tone mapping of event-based videos.

These algorithms can currently only be used with the ATIS, the only existing event-based grayscale camera. However, these algorithms are built on the AER protocol, which is widely used by existing neuromorphic vision sensors (in particular Lichtsteiner et al., 2008, see Delbrück et al., 2010 for a review); any future camera based on this protocol can benefit from our algorithms.

These results are the first of their kind since displaying asynchronous event-based vision sensors on conventional display devices (LCD screen, video-projector, printer, etc.) is a non explored problem till now. As long as no complete event-based and HDR imaging chain from the acquisition to display is available, there is a mandatory need to develop an event-based tone mapping algorithms to display as relevantly as possible such data on conventional systems for monitoring purposes, human-machine interaction, etc. Up to now, works on event-based visual data have mainly focused on processing the signal while displaying problems were not primary concerns. However, with maturing technology and techniques, event-based signal visualization may become increasingly interesting. This work is a first step to address this problem.

## Author contributions

CS, Designed experiments, performed analysis on all samples, interpreted data, and wrote manuscript. SI, Helped in data interpretation, to evaluate and edit the manuscript. CP, Helped to evaluate and edit the manuscript. RB, Helped in data interpretation, to evaluate and edit the manuscript.

### Conflict of interest statement

The authors declare that the research was conducted in the absence of any commercial or financial relationships that could be construed as a potential conflict of interest. The reviewer JM and handling Editor declared their shared affiliation, and the handling Editor states that the process nevertheless met the standards of a fair and objective review.
